# Commonalities in the lipid profile shift in ApoE ε4 risk variant and mild cognitive impairment highlight acyl chain remodeling

**DOI:** 10.1002/alz.087508

**Published:** 2025-01-03

**Authors:** Laura Beth McIntire, Jason Mares, Ana Paula Costa, Artur Lazarian, Krista Minéia Wartchow, William Dartora, Tal Nuriel, Vilas Menon

**Affiliations:** ^1^ Weill Cornell Medical College, Brain Health Imaging Institute, Department of Radiology, New York, NY USA; ^2^ Columbia University, New York, NY USA; ^3^ Weill Cornell Medicine, Brain Health Imaging Institute, Department of Radiology, New York, NY USA; ^4^ Weill Cornell Medicine, New York, NY USA; ^5^ Department of Cell Biology and Pathology, New York, NY USA; ^6^ Taub Institute for Research on Alzheimer’s disease, New York, NY USA; ^7^ The Taub Institute for Research on Alzheimer’s Disease and The Aging Brain, Columbia University, New York, NY USA; ^8^ Center for Translational & Computational Neuroimmunology, Department of Neurology, Columbia University Irving Medical Center, New York, NY USA

## Abstract

**Background:**

At least one‐third of the identified risk alleles from Genome Wide Association Studies of Alzheimer’s disease (AD) are involved in lipid metabolism, lipid transport, or direct lipid binding. BIN1 which is also known as Amphiphysin 2; and PICALM which are involved in phosphoinositide metabolism and binding rank just below the highest risk gene variant of Apolipoprotein E (ApoEε4), a cholesterol and phospholipid transporter. In addition to genetic variants, lipidomic studies have reported severe metabolic dysregulation in human autopsy brain tissue, CSF, blood and multiple mouse models of AD. We aimed to identify an overarching metabolic pathway in lipid metabolism by integrating analyses of transcriptomics and lipidomics in the Religious Order Study‐Memory Aging Project (ROS‐MAP) as well as models of disease.

**Method:**

Lipidomic data in ROS‐MAP was generated using the Biocrates AbsoluteIDQ p180 platform, a multiplexed targeted metabolomic assay covering lipids and metabolites including acylcarnitines, glycerophospholipids and sphingolipids. We confirmed global lipid dysregulation of acyl chain remodeling using pharmacological inhibitors of lipid modifying enzymes in cell models overexpressing amyloid precursor protein (APP) and identified similarly dysregulated lipids in an animal model of AD overexpressing APP harboring the Swedish mutation in a targeted lipidomic panel of over 600 lipid species.

**Result:**

Our analysis of transcriptomic data from ROS‐MAP lead to identification of multiple genes in the pathway for acyl chain remolding, Lands Cycle, which were associated with cognitive decline independently of amyloid and tau pathologies. Coordinate changes in lipids were found to be dysregulated in association with both mild cognitive impairment (MCI) and the ApoEe4 genotype which showed a correlated lipid profile shift. WGCNA analysis identified dysregulated lipids within a single module which are substrates and products in the Lands Cycle for acyl chain remodeling.

**Conclusion:**

Our studies highlight the critical dysregulation of acyl chain remodeling in ApoEe4 carriers and MCI in human brain which could be modeled in mouse and cell models of disease. A coordinated lipid profile shift in both ApoEe4 carriers and MCI suggest pathological changes in lipid metabolism underly early disease and highlight lipid dyshomeostasis as a tractable target for early disease modifying intervention.

